# Social work assessment of climate change: Case of disasters in greater Tzaneen municipality

**DOI:** 10.4102/jamba.v11i3.710

**Published:** 2019-07-02

**Authors:** Allucia L. Shokane

**Affiliations:** 1 Department of Social Work, University of Venda, Thohoyandou, South Africa

**Keywords:** Social Work, Disaster, Climate Change, Households, Vulnerability, Assessment

## Abstract

Climate-change-induced disasters such as floods, heavy storms, tornadoes and extreme lightning are becoming more frequent in Africa generally and in South Africa specifically. Several factors contribute to Africa’s high vulnerability to disasters, including the high rate of population growth, food insecurity, high levels of poverty, inappropriate use of natural resources and failures of policy and institutional frameworks. The study adopted an ecological systems theory as a theoretical framework to explain how social work in rural communities deals with climate-change-induced disasters. The aim was to explore and describe the role of social work in the assessment of climate change disaster predicaments. A qualitative approach, utilising an exploratory-descriptive design, was adopted for this study. A purposive sampling technique was used to select five social workers and two social auxiliary workers to participate in the study. Semi-structured interviews were applied in the research as a tool for data collection. Data were analysed qualitatively using thematic content analysis. The research concluded that social workers should intervene in climate-change-induced disasters by conducting assessments and providing disaster intervention strategies.

## Introduction

Climate-change-related events have far greater impacts on low- and medium-income countries than on high-income nations (UNISDR 2004). Cities in low- and medium-income countries will be more affected by such events owing to several interrelated hazard and risk factors to which low-income groups are exposed, as well as their pre-existing vulnerability (Downing & Dow 2006). Climate-change-induced disasters (floods, heavy storms, tornadoes and extreme lightning) are occurring more frequently around the globe and in South Africa.

Nhemachena et al. (2014) affirm that climate change impacts are already burdening most rural communities. Needless to say, no one is immune to these undesirable natural phenomena; therefore, by implication, the rural communities of Limpopo Province are obviously affected. Being in poverty and being subject to poor socio-economic factors and environmental factors, as contended by OFDA-CRED (2002) and ICSU (2005), they are the most adversely affected. Rubin (2011) further contends that poor and marginalised communities – who already live in stressed, dangerous and hazardous conditions – would potentially feel the worst effects of long-term changes in weather and increased extreme weather events. To this end, the effects of natural disasters are enormous, causing economic damage and loss of human capability (Collins 2009). The Intergovernmental Panel on Climate Change (2007) defines a natural disaster as:

[*A*] serious disruption of the functioning of a community or a society causing widespread human, material, economic and environment losses, which exceed the ability of the affected community or society to cope using its own resources. (p. 17)

In a 2011 study analysing legislation related to disaster risk reduction in South Africa, the International Federation of Red Cross and Red Crescent Societies (IFRCRC) reported that about half-a-million people per annum in South Africa are affected by natural disasters, which costs the state R12 billion. The natural disasters in South Africa have been dominated by localised incidents such as wildfires, seasonal flooding, hail, wind and snowstorms (IFRC 2011). According to Downing and Dow (2006), those who are most vulnerable to natural disasters and climate-related risks are poor people, whose livelihoods often directly depend on natural resources, which are unfortunately prone to climatic changes. The risk factors for people in rural areas who experience natural disasters are physical location, social and cultural aspects, education and information, as well as political will (Davids, Theron & Maphunye 2005).

### Background information

Studies indicate that it is imperative to recognise climate change as the foundation for disaster predicaments (Downing & Dow 2006; Green 2009; IPCC 2007). However, climate change is seldom discussed in mainstream social work (Dominelli 2011). Its first presentation on the world stage occurred in Copenhagen during the Conference of the Parties (COP15) in 2009 (Dominelli 2011, 2012). Climate change is described as ‘an on-going trend of changes in the earth’s general weather conditions because of an average rise in the temperature of the earth’s surface’ (Department of Environment Affairs 2011:8).

Evidence of rapid climate change comprises intense weather systems, with increased frequency of heavy rainfall and longer drought seasons (Green 2009; Winker 2010). Throughout history, the poor and marginalised have been the people most riskier and most vulnerable to the impacts of climate change (IPCC 2007). The vulnerability and risk factors weaken the abilities of communities to cope with the effects and stresses of climate change to which they are exposed (Chambers 2006; Green 2009; IPCC 2007; Tanner & Mitchell 2008). The impacts of climate-change-induced disasters are drought, floods or other natural disasters. This demands that various stakeholders, including social workers, contribute to disaster management practices.

### Problem statement

Communities are vulnerable to climate-change-induced disasters because of their demographic, cultural, historical or ecological characteristics (Zakour 1996). Therefore, this implies that the social work profession has an inherent role to play in supporting communities affected by natural disasters around the globe. Equally, like all other stakeholders, social workers are required to respond appropriately to the disaster impacts in order to address these adverse effects of climate-change-induced disasters. This has been asserted by the International Association of Social Workers (IASSW) and International Federation of Social Workers (IFSW) by describing social work as ‘a practice-based profession and an academic discipline that promotes social change and development, social cohesion, and the empowerment and liberation of people’ (2014:1). In addition, the IASSW and IFSW (2014) affirm that social work should engage with people and relevant structures to address life challenges to enhance people’s well-being. Furthermore, Nicholas, Rautenbach and Maistry (2010) attest that social work should address factors that make communities vulnerable to natural disasters.

There is, however, unfortunately, a dearth of research related to social work disasters in South Africa (Dominelli 2012; Gray, Coates & Hetherington 2012; Molyneux 2010; Zakour 1996; Zapf 2009). This has been evident in recent literature in social work disaster research that social work should take a lead in creating a new vision for practice in dealing with disasters (Besthorn 2012; Coates 2005; Dominelli 2012; Molyneux 2010; Zapf 2009). The researchers in this field call for a paradigm shift among the professionals, developing ecological consciousness among social work scholars and practitioners to appreciate environmental social work as an essential but underdeveloped area of professional scholarship and practice (Besthorn 2012; Coates 2005; Dominelli 2012; Zapf 2009). Furthermore, scholars in social work (Bhaskar et al. 2010; Winker 2010) appeal for research that integrates the work of biophysical and social sciences in disaster management.

In most cases, in spite of social work centrality in the normal functioning of people, social workers are side-lined and considered only after the damage has been caused. If involved, they are required to perform miracles and be welfarists by distributing food parcels and other basic amenities. Social workers, in times of climate-change-induced disasters, can contribute positively by conducting multidimensional assessments, which can be of help for their intervention to incite responsibility in other stakeholders during such a critical and painful time. Additionally, this will be in line with the purpose of social work, which is to enhance people’s social functioning and empowering people affected by natural disasters through all stages of assessment, rescue, planning and intervention for recovery and preparedness (Dominelli 2011; Tang & Cheung 2005). Although social workers are responsible for conducting assessments on victims’ households, the key focus of their function is to render services to alleviate the impact of natural disasters on the livelihoods of people and increase their resilience capabilities. It is against this background that this article addresses the role of social work in disaster interventions.

### Social work assessment process

Natural disasters are inevitable and occur throughout the world, and many social workers and students should engage in disaster relief processes (Tang & Cheung 2005). In this article, I argue that the role of social work assessment is essential in disaster management. Social work practice is considered by Compton, Galaway and Cournoyer (2004) to be the professional application of social work values, principles and techniques. This is aimed at helping people to obtain social work services, such as counselling and therapy, to improve their social functioning. However, before such social work services can be rendered, a proper assessment is essential. Assessment in social work is described by Coulshed and Orme (2012) as an ongoing participatory process that seeks to understand the service user and his or her situation and sets a basis for planning how change or improvement can be achieved. In addition, Coulshed and Orme (2012) and Compton et al. (2004) assert that social work assessment is the basis for planning what needs to be done in order to bring about the required change.

Social workers can fulfil many roles in the wake of a national disaster because they have the expertise to address many social issues people face (Zakour 1996). In addition, social workers may provide basic professional intervention, help reduce tension and contain unpredictable reactions (Yanay & Benjamin 2005). Dominelli (2012) upholds that social workers can be involved in assessing the aftermath of natural disasters and developing plans for the distribution of aid, as well as re-uniting families and participating in long-term development.

Social work assessment is based on five guiding principles, proposed by Milner and O’Brien (2009), which help clarify and direct practice in all areas of social work disaster interventions. The five guiding principles, according to Milner and O’Brien (2009), underlying social work assessments and which can be applied in disaster interventions are presented as follows:

#### Understanding the need principle

The principle of need orientates the social worker towards exploring and understanding the service user’s situation (Coulshed & Orme 2012). In the context of this article, it is, however, important for the people affected by natural disasters to be able to identify their own needs after experiencing a disaster.

#### Systems and ecology principle

The central aspect of working with systems and ecology principle draws on the fact that households that are affected by natural disasters are part of systems, networks and connected to other people and communities (Milner & O’Brien 2009). This is mostly about how the impacts of natural disasters have an influence and effect on others in the community.

#### Building on strengths principle

It is a well-known fact that natural disasters leave their victims hopeless and destitute. The principle of the strengths in assessment process must take account of the capacities, strengths and protective factors of the people affected by natural disasters. This could be done through applying assets-based approaches that seek to recognise their resilience. Shokane (2016:107) affirms that when resilience is applied in the context of climate-change-induced disasters, it ‘seeks to discover uniqueness and indigenous ways of coping and guides that practitioners can use to intervene in a culturally appropriate way’.

#### Being person-centred principle

The fourth principle is that the assessment of climate-change-induced disasters should focus on a person-centred approach, which involves direct interaction with the person affected by natural disasters in order to determine a suitable disaster intervention, which is informed by theoretical and knowledge base underpinning practices. In short, this principle ensures that no assessment in disaster social work should lose sight of the needs of a person or a household affected by natural disaster. In line with social work’s values, the involvement of service users in decisions about their own situation is paramount.

#### Taking an Interdisciplinary approach principle

In any disaster, intervention is of utmost importance to take an interdisciplinary approach. This is because different professionals have particular areas of expertise in disaster interventions. Coulshed and Orme describe an interdisciplinary collaboration as ‘an effective interpersonal process that facilitates the achievement of goals that cannot be reached when independent professions act on their own’ (2012:5). Professionals from different disciplines, comprising life-saving and medical personnel, also join forces with social workers in managing natural disasters (Tang & Cheung 2005). The multi-disciplinary team usually analyses the needs of those affected depending on the different degrees of exposure to the natural disaster, as this determines the intervention strategies followed by the analysis (Collins 2009).

#### Theoretical framework

The ecological systems theory was appropriate in assessing and describing the role of social work in the assessment of households affected by climate change. A theoretical framework in this research study was of prominence as it supported the researcher with the data-gathering process at a household level. The ecological systems theory is concerned with how individuals and the environment achieve an adaptive balance and also why they sometimes fail to achieve the balance (Zastrow 2010). This framework was applied to understand how social workers intervene in disasters in rural communities in addition to withstanding and dealing with climate-change-induced disasters. The ecological systems theory affirms that individuals are situated ‘within layers of systems from immediate family up to wider society’ and any assessment is required to take account of these layers of connections and influences (Coulshed & Orme 2012:6).

Furthermore, the assessment of social work should determine the ability of communities to cope and recover from stresses and shocks that are induced by disasters in order to ensure a proper intervention strategy (Collins 2009; Green 2009). This terminology is increasingly utilised in disaster reduction. As natural disasters affect communities differently, according to their respective vulnerabilities, assessments must be locally specific and appropriate to the context (Vincent, Naess & Goulden 2013).

The ecological systems theory places great emphasis on involving people in both the identification and the implementation of any disaster programme activities and interventions (Rakodi 2002; Zastrow 2010). The objective of this article is to explore the role played by social workers in the assessment of natural disasters in order to ascertain the proper development of intervention strategies for social work disaster management in rural areas. Therefore, the ecological systems theory assisted in addressing and describing the role of social work in climate-change-induced disaster interventions.

## Research method and design

A qualitative, exploratory, descriptive design was implemented to describe the role of social work in the assessment of households affected by climate-change-induced disasters (Creswell 2009; Monnette et al. 2014). The qualitative design was absolute in providing rich information from participants’ perceptions and experiences (Babbie & Mouton 2011). The assessment of the role of social work in disaster interventions was carried out at Naphuno subdistrict, a community affected by natural disasters approximately 30 km outside Greater Tzaneen Municipality in Limpopo Province. The researcher’s selection of the community was based on prior knowledge in having been a practising social worker at the Department of Social Development. Furthermore, the researcher had a desire to investigate the role of social work in climate-change-induced disasters, as ultimately, social workers are expected to provide social relief of distress services in disaster interventions. The researcher put together a small sample of five social workers, two social auxiliary workers and 10 households who had been affected by natural disasters. These individuals from the 10 households were assessed by social workers following a disaster and were purposively selected so that comprehensive knowledge on the topic under study could be yielded (Babbie & Mouton 2011).

### Data collection and analyses

Qualitative methods of data gathering, such as semi-structured interviews, were utilised to collect information in order to obtain their experiences on a specific set of open questions concerning their disaster interventions from social workers. Of 10 households, a focus group discussion among seven members was conducted, and three one-to-one in-depth interviews were held. The data were collected in November 2015. The following themes were discussed, which guided the interviews: climate change’s impact in inducing natural disasters, and the role played by social workers in disaster intervention.

The data collection methods assisted in providing answers to the following research questions for the study:

- How do social workers intervene in communities affected by natural disasters?
- What is the role of social work in assessing community disaster situations?

The responses were recorded, counted and transcribed, and then translated into English as interviews were conducted in local language (namely, *Sepedi,* also known as *Northern Sotho*). Qualitative thematic content data analysis, which was adapted from Creswell (2009), was applied to analyse the collected data. The collected data were transcribed for analysis. The data were processed and reduced to manageable parts. The data were coded in order to analyse and make sense of it. The large quantities of data were organised into much fewer categories in order to determine trustworthiness (Lincoln & Guba 1999). Data display was utilised to draw conclusions about qualitative materials collected through semi-structured interviews, and necessary actions were taken.

### Ethical consideration

The following ethical issues were considered throughout the study. Permission to conduct the study was obtained from the University of Venda Publication and Research Committee.

## Discussions of findings

The following two main themes emerged during our analysis: the involvement of social workers in assessment of climate-change-induced disasters and the role played by social workers in the assessment of natural disasters.

### Theme 1: Assessment of natural disasters

The findings from the analysed data have shown that social workers are involved in the assessment of communities affected by natural disasters. The social workers who were invited confirmed that they conduct assessments in households affected by a natural disaster. In substantiation, they narrated the following:

After a household has been affected by a natural disaster, we conduct an assessment, which is a type of screening, to assess whether the household is destitute or not as a result of a natural disaster.

We are usually notified by the members of the community – such as, counsellors, community development workers and even affected community members themselves.

Once damage that has been caused by a natural disaster has been confirmed, we recommend the provision of social relief of distress in the form of food parcels (containing basic food necessities), blankets and cash.

In extreme cases of damage, we place the people affected by the natural disaster in alternative accommodation such as churches and community halls. (Social workers, pers. comm., n.d.)

The findings confirmed that social workers conduct their assessment based on the Department of Social Development’s (DSD) intervention strategy of providing social relief of distress to people affected by natural disasters. The DSD intervention strategy is primarily based on the immediate needs of people affected by a natural disaster through its Social Relief of Distress (SRD). The SRD allows social workers to assess and provide food parcels and vouchers to buy food for the people affected by a natural disaster. The findings are similar to the research conducted by Makiwane and Rama (2004), who stated that:

The SRD strives to provide relief to individuals who experienced socio-economic distress resulting from a range of circumstances such as a disaster, or communities that have been declared a disaster area. (p. 5)

The finding confirms that social workers conduct assessments in order to address the community’s vulnerability and level of destitution as a result of a loss of income and property caused by the natural disasters as depicted in Table 1.

**TABLE 1:** Assessment of disasters.

No of social workers	No. of households assessed by social workers	Types of natural disasters assessed	Outcome of assessment	Response
Social work 1	10	Rain or floods (*pula*); windstorms (*ledimo*) and lightning (*legadima*).	Not coping.	Social relief of distress.
Social work 2	10	Rain or floods (*pula*).	Not coping.	Food parcels.
Social work 3	8	Rain or floods (*pula*).	Not coping.	Cash vouchers.
Social work 4	9	Rain or floods (pula), windstorms (*ledimo*) and lightning (*legadima*).	Not coping.	
Social work 5	13	Rain or floods (*pula*).	Not coping.	Referral to SASSA

**Total**	**50**			

Table 1 indicates a significant number of social workers (*n* = 5) reporting that they had conducted social work assessments on households who had experienced shocks to climate-change-induced disasters. This was done to assess whether a person who is affected by a natural disaster is able to cope and manage after he or she has experienced a disaster.

The findings of the study revealed that the majority of the participants (*n* = 5) indicated that the types of natural disasters assessed were pertinent to climate-change-induced disasters. The social work assessment was conducted owing to various damages to property and livelihoods attributable to rain or floods [*pula*], windstorms [*ledimo*] and lightning [*legadima*]. The 50 households assessed by social workers reported that they had experienced the shock of natural disasters between 2012 and 2014. The duration of recovery from various disaster shocks took place over 2 months. The three most significant disaster shocks experienced by households were as follows: people had their property struck by lightning; their corrugated roof was blown away by hail and windstorms; and their crops damaged by floods. The findings are consistent with the findings of Zapf (2009) in social work disaster research, indicating that social work disaster services include assessing and helping people to qualify for getting aid for home reconstruction and the replacement of other material losses.

The social work assessment’s findings have indicated that the extent of damage has resulted in some of their houses being demolished or the roof blown away by the windstorm. The social workers revealed that, after a disaster, almost all the assessed households (*n* = 50) could not manage repairing their household. The social workers responded by providing social relief of distress to the people affected to enable them to cope with their situation. Although they could not provide them with the required building materials, they were referred to the South African Social Security Agency (SASSA) to apply for a temporary social grant with a social worker’s motivational report.

### Theme 2: Social workers assessments

The second theme emerged from the analysed data of the 10 participants from households affected by natural disasters. The participants provided diverse, but intense, descriptions indicating their experiences of climate-change-induced disasters and their vulnerability to these, including the role played by social work in such interventions. The participants remarked on the level of support received from social workers after experiencing natural disasters. The important coping strategy after a climate-change-induced disaster occurrence was threefold: having support from the social workers; the provision of food parcels; and the intervention of SRD. The participants indicated that the estimated total value of loss after a disaster is not quantifiable. In addition, sadly, some participants indicated that social workers would often only assess the disaster and never return to the community to render the required further assistance or relief. The findings collected from the participants (*n* = 10) from the affected households are depicted in Table 2.

**TABLE 2:** Social workers assessments.

No of participants who were assessed by a social worker	Role of the social worker	Level of response
*N* = 10	Assessment and collection of data from people affected by natural disasters; Applying professional knowledge (theory, skills and practice); Intervention: provision of counselling and material assistance; Recommendation and referral; Social Relief of Distress (SRD).	Counselling and support provided; SRD provided; Complained that relief ‘took too long’; SRD grant either in sufficient or provided late.

Table 2 presented the findings collected from the participants from the affected households, confirming the fact that social workers did conduct assessments and collect information after their having experienced a natural disaster. However, they indicated that sometimes social workers take long to respond with the required material assistance. Furthermore, they indicated that when the social workers did eventually respond, they would deliver cash vouchers and blankets six months after a disaster had occurred. The following extract from the focus group discussions demonstrates this view:

Yes, the social workers comes to visit us after a disaster to check the extent of damage.

However, some social workers disappear and come back 6 to 8 months after the disaster has occurred.

They will only give you money to assist to rebuild the damaged house property. We have received the money from social workers, but since it is long after the disaster has occurred, we use the money for something else.

Most of the participants from the assessed households (*n* = 10) indicated that they support the proposal that social workers should be involved in the intervention of people affected by natural disasters. The participants indicated that social workers understood them when they cried for help. This finding is similar to the research conducted by Thabede (2008), which asserts that the helping professionals should understand African socio-cultural beliefs in order to appreciate what informs the behaviours of African clients.

## Implications for practice

The study envisages providing clarifications on the role of social work in disaster management. This article argues that the role and involvement of social workers in climate-change-induced disasters is necessary (Jones & Truell 2012). Social workers need to understand the social, cultural, economic, political and historical contexts of the locality in which the disaster occurred in order to intervene appropriately (Gray et al. 2012). The research will contribute to the problem-solving knowledge of social work in intervening in communities affected by natural disasters. This will be in line with the Global Agenda for Social Work and Social Development’s commitment to work towards environmental sustainability in responding to the impacts of natural disasters by conducting research in social work in cooperation with communities (Jones & Truell 2012). The research output will assist policy-makers, such as national DSD, in planning and assessment for future climate-change-induced disasters.

## Limitation to the study

The study only focused on selected social workers who were involved in the assessment of people affected by natural disasters and in a specific area in Greater Tzaneen Municipality.

## Conclusion

The purpose of carrying out social work assessments in any interventions, including disaster interventions, is usually to identify levels of need or risk or to form an understanding when making initial contact with a person affected by natural disaster. This study assessed the role performed by social workers in assessing households affected by climate-change-induced disasters in rural areas. The climate-change-induced disasters reduced the abilities of some communities or households to cope with the events and stresses to which they were exposed, which required a social work intervention. The study has also ascertained that a household’s encounter with a natural disaster is also a social problem that requires a social work intervention. In addition, the findings revealed that the assessment of impacts of natural disasters is important to guide proper social work intervention. Furthermore, by developing and applying integrated (multi-level intervention) approaches that incorporate established interventions, social workers can distinctly improve intervention services for clients and their families (Makhubele 2013:9).

## Recommendations

The social worker’s assessment is essential for collecting and disseminating information and assessing the severity of a given situation. Having chosen a framework for assessment in disaster interventions, the social worker should include an awareness of social work theories; knowledge of assessment; and principles of assessments. Of utmost importance in any assessment that involves interdisciplinary collaboration, the information collected should be integrated with the professionals who are also intervening in the disaster, although it could be stored separately. The social work profession has an important role to contribute to disaster research and practice. The outcome of the study recommends that social work interventions dealing with natural disasters should be developed in a participatory way in cooperation with the affected communities.
